# The Hospitals, &c. of New York

**Published:** 1859-09

**Authors:** 


### EDITORIAL DEPARTMENT.

---

### *The Hospitals, etc., of New York.*

Under this caption we design to supply our readers, in successive numbers of our Journal, with a brief account of the different hospitals of New York, and other institutions for the relief of persons suffering from disease. The profession at large are but imperfectly acquainted with the charitable provisions for the sick existing in this city, and the resources which these afford for clinical instruction. We are led to form this conclusion from our own inadequate knowledge with regard to the subject prior to our removal to the city, and by the apparent want of information among medical men with whom we have been brought into contact. The remark applies, of course, more especially to members of the medical profession residing out of the city; but measurably, also, to the profession within the city. As proof of the correctness of the conclusion, we may instance the number of medical students attracted here by the advantages for prosecuting medical studies. The number, it is true, is by no means insignificant; but that it is not much greater than it is, may fairly be considered as evidence that the opportunities for clinical study are not fully understood, and, consequently, not sufficiently appreciated. An acquaintance with the institutions referred to is desirable, not only as information intrinsically interesting to physicians, but as connected, in various ways, with the interests of medical education. An increase of the number of students in medicine coming to New York for instruction, would, of course, enhance the prosperity of the medical schools; but, assuming that superior advantages are to be here enjoyed, the students, not less than the schools, and thereby the profession, are benefited by the election of this city as a field of study. Moreover, clinical study and teaching affect, in no small measure, the character and usefulness of charitable institutions, irrespective of medical education. The investigation of disease, with reference to the acquisition or diffusion of knowledge, redounds to the advantage of patients, as well as the promotion of science, directly, by securing greater interest in, and a more thorough acquaintance with, individual cases, and, indirectly, by contributing to progressive improvement in the art of healing. Medical education is a matter of such importance to the public welfare, that its interests should always be considered in the organization and management of charitable institutions for the relief of the sick; and it is a happy circumstance that proper attention to its interests tends to promote the more immediate objects of philanthropy to which these institutions are devoted. Progress in the science of medicine and efficient relief, are mutually subservient to each other; and that institution is incompletely constituted, in which ample provisions for scientific study and teaching are deficient. Hence, in noticing the hospitals, etc., of New York, it will be pertinent to inquire whether the resources which they afford for medical instruction are sufficiently developed and improved.

We propose to notice, in this number, the institutions for the relief of sick children. The institutions falling under this head are the following: The "Nursery Hospital," on Randall's Island; the "Colored Home," the "Colored Orphan Asylum," and the "Nursery and Child's Hospital."

Nursery Hospital, Randall's Island.—It was a happy thought to appropriate the trio of beautiful islands in the East River to public institutions. These islands are neither too small nor too large, for this purpose; they are sufficiently isolated, both from the city and country, and are conveniently accessible; the situation is salubrious, and the soil fertile. The farthest from the city of the series, is Randall's Island; and here are established the nursery department of the Alms House, and the House of Refuge for Juvenile Offenders.

The nursery department of the Alms House receives children of both sexes from between the ages of two and fifteen years. A certain number, in due time, are discharged for indenture, some are adopted, others are returned to friends; and those who, from imbecility or disease, are unfitted for indenture, after the age of nineteen, are transferred to other departments of the Alms House. The children are well cared for, taught the elementary branches of common schools, and, if able, at a proper age are made to give a portion of the day to some appropriate mechanical occupation. They are well fed, comfortably clothed, present a tidy appearance, and seem to be happy. Our present business, however, is with the sick that are received into the nursery hospital. From the Report of the Physician to the Governor of the Alms House for 1858, it appears that during the year the whole number of children under treatment was 1893. Of this number, there were discharged, 1489; died, 142; and there remained in hospital at the end of the year, 262.

The report just referred to contains a table of the diseases treated during the year. On looking over the list, we find that it contains the diseases common to all ages, with a preponderance of those to which childhood is most liable. Conjunctivitis was the most frequent, 210 cases occurring during the year. Of scrofulous ophthalmia, there were 72 cases; and of purulent ophthalmia, 105 cases. There were over 70 cases of pneumonia, and 25 cases of capillary bronchitis. Over 50 cases of fever occurred, ephemeral, typhoid, and periodical, and nearly 200 cases of rubeola; the latter presenting a variety of complications. The cases of caries of the vertebræ numbered 17; of *morbus coxarius*, 19; of meningitis, 10; of cancrum oris, 9; of chorea, 8; of ulcerative stomatitis, 30, etc. These citations from the report suffice to show that this hospital offers great advantages for the study of the diseases of children. It is not, however, at present made subservient to clinical teaching. For this end, regular courses of lectures should be instituted, to which medical students and practitioners should have free access. This is to be desired the more, because the opportunities for studying the diseases of children at the bed-side, during medical pupilage, can exist only in large cities, and nowhere in this country to the same extent as in the city of New York. The truth is, a large proportion of students in medicine graduate and engage in practice with but little, if any, practical knowledge of these diseases. They find themselves at a loss in the diagnosis of even the most common affections. How often, for example, is rubeola confounded with scarlatina; and *vice versa!* The uncertainty and errors growing out of this want of knowledge to be acquired only at the bed-side, incapacitate for the proper management of cases of disease, and for success in obtaining practice. We hope to see the Nursery Hospital at Randall's Island become a school for clinical instruction like the *Hôpital des Enfans*, of Paris. This is to be done, not by mere casual observations at the bed-side, valuable as these may be, but by providing, in addition, for regular systematic courses of lectures.

The nursery institution on this Island embraces a ward for idiotic children, which contains, at the present time, over thirty inmates, and offers a fine field for the study of the different forms and degrees of idiocy; a subject of much interest, and of practical importance with reference to means for improving the condition of this afflicted class of human beings.

The visitor of the Island cannot fail to be struck with the evidence, on all sides, of the judgment and care of the medical management of the children; which has been for many years confided to Dr. Whittelsey, aided by the resident assistants. To Dr. Crook, one of the assistants, our thanks are due for his courtesy in showing and explaining all the details of the institution.

The House of Refuge for Juvenile Offenders, situated on this island, a State institution, is also under the medical charge of Dr. Whittlesey. Of its several hundred inmates, however, the number on the sick report does not average more than six; a fact which speaks well for the regulation of the establishment, in a hygienic point of view.

Colored Home and Colored Orphan Asylum.—These institutions are under the supervision of the Governors of the Alms House. The Colored Home embraces a lying-in department and nursery hospital. During the last year, 87 children were born or received at this institution. There were 8 deaths under 10 years of age. As regards the relief of sick children, the Colored Orphan Asylum is much more extensive. In the latter institution, for the year ending December, 1858, there had been 118 cases of sickness, and 10 deaths; in 8 of the latter, the disease being phthisis.

The opportunity of observing diseases as manifested in the African race, is of considerable importance to the medical student, especially if he designs to practice in the South. This remark applies particularly to the eruptive fevers; and at the Colored Orphan Asylum, during one year, there were 74 cases of scarlatina, and 34 cases of rubeola. These institutions should be accessible to students, for this object, during stated hours on certain days of the week.

Nursery and Child's Hospital.—This is a private institution, situated on 51st Street, near the 3d Avenue, and is under the direction of a board of lady managers. A commodious building has recently been erected. From the fifth annual report, it appears that during the year ending March 1st, there had been received into the institution 195 women and 427 children. Among the children, during the year, there were 373 cases for hospital treatment.

Children are received at this institution from the hour of birth. The objects are, to provide a home for children who are homeless, or who cannot be properly cared for at home, and a hospital for sick children who cannot receive proper attention at their homes. In addition, wet-nurses are received, and when their services are not needed at the institution, they remain until they obtain situations in private families. A dispensary is connected with the institution, where children are examined and prescribed for daily at stated hours, medicines not being furnished. The institution is supported by subscriptions, voluntary contributions, and a moderate payment for the board of each child. The wards are spacious, airy, and clean. Two children are assigned to each wet-nurse, and they sleep in a single crib by the side of her bed. Both children and nurses are tidy, and the latter generally present a healthy appearance. Children are received for a longer or shorter period, for a single day even; so that this institution may serve the same purpose as the Parisian *Crèche.* Many of the children are puny and sickly; but this is to be expected in view of hereditary affections, and the circumstances unfavorable to health preceding their admission. The objects of the institution are most commendable, and should meet with the warm sympathy and active co-operation of the philanthropic. The medical board recommend efforts to obtain more space for exercise in the open air, and a place in the country for convalescents, and for the refuge of the feeble in the summer months. These improvements will undoubtedly add much to the efficiency of the institution. The medical board consists of four attending physicians, four consulting physicians, and a resident physician. To the courtesy of the latter, Dr. Hadden, we are indebted for an opportunity of examining personally the details of the establishment.

This hospital affords an excellent field for clinical instruction. It supplies a deficiency which exists at the nursery hospital on Randall's Island, viz., the opportunity of observing diseases of children under two years of age. Children are not received under this age at Randall's Island. The report by Dr. Geo. T. Elliot, jr., on behalf of the medical board, states that "a systematic course of lectures is in operation, by which, for the first time in this city, the diseases of infancy have been made the subject of bed-side lectures." The steps toward clinical teaching, however, as we learn, have been, as yet, but initiatory. It is to be hoped that they may lead the way to the improvement, to the fullest extent, of the resources afforded in this and other institutions of New York, for practical instruction in the diseases of children. These resources have but to be improved to be appreciated. It is not for lack of resources that the medical student, in estimating the advantages of the city of New York, does not take into account facilities for studying this important class of diseases, such as no other city on this continent can offer.*

